# Neurobiology of Treatment-Emergent Mania Associated with Antidepressant: A Narrative Review Update

**DOI:** 10.1007/s11920-026-01727-8

**Published:** 2026-11-14

**Authors:** Jefté Peper-Nascimento, Taise Possamai-Della, Luciane B. Ceretta, João Quevedo, Samira S. Valvassori

**Affiliations:** 1 Laboratório de Psiquiatria Translacional, Programa de Pós-Graduação em Ciências da Saúde, Universidade do Extremo Sul Catarinense (UNESC), Criciúma, SC Brazil; 2 Programa de Pós-Graduação em Saúde Coletiva, Universidade do Extremo Sul Catarinense (UNESC), Criciúma, SC Brazil; 3 Center for Interventional Psychiatry, Faillace Department of Psychiatry and Behavioral Sciences, McGovern Medical School, The University of Texas Health Science Center at Houston (UTHealth Houston), Houston, TX USA

**Keywords:** Bipolar disorder, Mania, Hypomania, Antidepressants, Treatment-emergent mania

## Abstract

**Purpose of Review:**

Bipolar disorder (BD) is a complex and debilitating condition in which antidepressant-associated treatment-emergent mania (TEM) poses a significant clinical challenge. This narrative review synthesizes current insights into the neurobiological mechanisms underlying this phenomenon, aiming to update the understanding in the field.

**Recent Findings:**

Clinical guidelines often caution against antidepressant monotherapy due to the risks of mood destabilization and switching, particularly in BD type I. A review of specific antidepressant classes reveals that tricyclic antidepressants (TCAs) carry a high risk of TEM, which has been linked to disruptions in cholinergic-adrenergic balance and heightened noradrenergic activity. Selective serotonin reuptake inhibitors (SSRIs), while less implicated than TCAs, are also associated with TEM, involving serotonin neurotransmission and potential interactions with muscarinic and histaminergic receptors. Furthermore, evidence suggests that even atypical antidepressants with non-monoaminergic profiles can induce manic switches, highlighting the vulnerability of some individuals and the influence of genetic predispositions. In addition, the effects of antidepressants associated with TEM can also be influenced by factors related to hepatic metabolism, as well as alterations in mitochondrial activity and immunoinflammatory processes, which can modulate the response to treatment and vulnerability to mood dysregulation.

**Summary:**

Antidepressants are one possible precipitant of TEM, but the phenomenon is better understood as a vulnerability-dependent outcome rather than a universal consequence of antidepressant exposure. Current evidence suggests that TEM may arise from neurobiological perturbations that are partially distinct across antidepressant classes and interact with individual susceptibility factors. These findings point to a broader network-level dysregulation of mood that extends beyond isolated neurotransmitter systems. These observations reinforce the importance of personalized treatment strategies that prioritize mood stabilization, careful patient selection, and close clinical monitoring when antidepressants are used in bipolar depression.

## Introduction

Bipolar disorder (BD) is a debilitating condition characterized by a complex clinical landscape and significant cognitive and functional impairments that affect the quality of life of both patients and their families [1, 2]. Its symptomatology encompasses a spectrum of pathological mood swings, including euthymia, depression, mixed episodes, and the hallmark occurrence of mania and/or hypomania [3]. Mania is defined as a distinct period of at least one week of abnormally elevated, expansive, or irritable mood accompanied by increased goal-directed activity, grandiosity, decreased need for sleep, and risky behaviors [4, 5]. In contrast, hypomania presents as a less severe manifestation lasting at least four consecutive days; while it shares similar symptoms with mania, it typically does not require hospitalization or cause severe functional impairment, though it still involves significant behavioral changes that contrast sharply with the profound sadness and anhedonia characteristic of depressive episodes [4].

The term “switch mania,” a hallmark phenomenon of BD, refers to the abrupt transition from a depressive or euthymic state to a manic episode [5]. This rapid shift in mood and behavior can be disruptive and challenging for individuals with BD, as well as for their families and healthcare providers [6]. Switch mania is characterized by a sudden onset of elevated mood, increased energy, impulsiveness, and decreased need for sleep, among other symptoms typical of manic episodes [7]. Triggers for the manic switch in individuals with BD can vary widely and are often individual-specific.

It is well described in the literature that some antidepressant medications, commonly prescribed for the treatment of depressive symptoms in BD, have been associated with the risk of triggering manic episodes or accelerating cycling between mood states, a phenomenon reported as treatment-emergent mania (TEM). Studies show that TEM occurs in 36–38% of patients exposed to antidepressants [8, 9], with the majority of cases occurring during antidepressant monotherapy [8]. This underscores the delicate balance required when treating BD with pharmacotherapy [10]. This phenomenon supports the theory that the mania switch may be linked to alterations in neurotransmission systems, particularly given that most antidepressants function by inhibiting serotonin reuptake [11]. Other changes in medication, including starting or stopping mood stabilizer drugs without proper supervision, can also destabilize mood and lead to a manic switch [12].

Managing TEM in individuals with BD often requires a comprehensive treatment approach that may include mood stabilizers, antipsychotic medications, psychotherapy, and lifestyle modifications. Early detection of prodromal symptoms and close monitoring by mental health professionals are essential for preventing or minimizing the impact of switch mania episodes [13]. By deepening their understanding of the neurobiological underpinnings of TEM, researchers and clinicians can develop more precise and efficient interventions to help individuals with BD effectively manage their symptoms and enhance their quality of life. Therefore, this literature review aims to synthesize current insights into TEM, with a primary focus on the underlying neurobiological mechanisms. All studies included in this review are summarized in Table 1 and 2.

**Table 1:** ​Summary of preclinical studies on treatment-emergent mania​​

Authors and year	Title of paper	Methodology		Behavioral Effects	Molecular Effect
		Animal Model	Drugs		
Valvassori et al., 2022 [32]	Imipramine induces hyperactivity in rats pretreated with ouabain: Implications to the mania switch induced by antidepressants	Animal model of mania induced by OUA. Wistar Rats	Imipramine (10 mg/kg) was administered once a day between 9 and 14 days after OUA administration.	**OUA administration** ↑ Manic-like behavior (day 7) ↑Depressive-like behavior (day 14) **Imipramine administration** ↑ Mania switch-like effect (day 14)	**OUA administration** ↑ Oxidative damage in the brain (4-HNE, carbonyl). ↑ Activity of antioxidant enzymes in the brain (GPx, GR). **Imipramine administration** Potentialized the oxidative damage of OUA. No significant differences between groups were observed in the Na+/K+-ATPase activity.

*4-HNE*​ 4-hydroxynonenal, *GPx*​ glutathione peroxidase, *GR*​ glutathione reductase,*ICV*​ intracerebroventricular injection, *Na⁺/K⁺-ATPase*​ sodium-potassium ATPase, *OUA* ouabain. ↑ Increase in the parameter; ↓ Decrease in the parameter

**Table 2:** Summary of clinical studies on treatment-emergent mania

Authors and year	Title of paper	Methodology		Trigger	Clinical Outcomes	Molecular Effect
		Type of study	Population			
Thorpe et al., 2014 [47]	Agomelatine-associated manic switch in bipolar depression: A case report	Case report	31-year-old female	Agomelatine at a dose of 25 mg	**Two months after treatment** ↑ Confidence, pressure of speech, episodic irritability, an irregular eating pattern, and reduction in sleep to four hours per night. ↑ Cleaning and spending behaviors.	Not applicable
Bhowmik et al., 2014 [22]	Risk of manic switch associated with antidepressant therapy in pediatric bipolar depression	Cohort study (2003–2007)	Children and adolescents (ages 6–18 years) with diagnosis of BD (*N* = 4,147)	AD monotherapy Second-generation antipsychotic monotherapy AD polytherapy	**AD monotherapy and polytherapy** ↑ Risk of manic switch. Higher risk of manic switch associated with antidepressant monotherapy than with SGA monotherapy.	Not applicable
Viktorin et al., 2014 [23]	The risk of switch to mania in patients with bipolar disorder during treatment with an antidepressant alone and in combination with a mood stabilizer	Observational Study	Patients with BD (*N* = 3,240)	AD monotherapy (Main types; Citalopram, Sertraline, Mirtazapine) AD plus a mood stabilizer	**AD monotherapy** ↑ Risk of mania within the first 3 months of treatment. Did not find an increased risk of manic episodes in the short or longer term for patients treated with an AD and a concurrent mood stabilizer.	Not applicable
Patel et al., 2015 [30]	Do antidepressants increase the risk of mania and bipolar disorder in people with depression? A retrospective electronic case register cohort study	Cohort study /observational study (2006–2013)	Adults with unipolar depression (*N* = 21,012)	AD therapy (electronic health records)	The incidence rate of mania/bipolar disorder was 10.9 per 1000 person-years. **AD treatment** ↑ Incidence of mania/bipolar disorder ranging from 13.1 to 19.1 per 1000 person-years. Multivariable analysis indicated a significant association with selective serotonin reuptake inhibitors and venlafaxine.	Not applicable
Niitsu et al., 2015 [13]	Predictors of switch from depression to mania in bipolar disorder	Multi-center and naturalistic cohort study	Patients with BD *N* = 1720	Fluoxetine, sertraline, paroxetine, quetiapine or clozapine	**SSRIs** ↑ Risk of switch was associated with history of switch induced by fluoxetine, sertraline and paroxetine. **Quetiapine and clozapine** ↑ risk of switching.	Not applicable
Tada et al., 2015 [26]	Antidepressant dose and treatment response in bipolar depression: Reanalysis of the Systematic Treatment Enhancement Program for Bipolar Disorder (STEP-BD) data	Randomized Controlled Trial	Patients with BD (*N* = 333)	Adjunctive AD (Paroxetine, bupropion) Groups: high-dose (*n* = 102); low-dose (*n* = 62); placebo (*n* = 169).	No significant associations between the adjunctive AD dose status and the risks of treatment-emergent affective switch Subjects with concomitant manic symptoms or those with a diagnosis of bipolar I disorder at baseline are more likely to experience treatment-emergent affective switch.	Not applicable
Gorwood et al., 2016 [27]	The number of past manic episodes is the best predictor of antidepressant-emergent manic switch in a cohort of bipolar depressed patients	Cross-sectional study	Patients with bipolar depression (*N* = 1242. Patients were receiving mood stabilizers for at least 3 months, and were treated with AD therapy, or were being switched to a new AD treatment.	Atypical (Mianserine, Mirtazapine & Tianeptine) (75.58%), SSRI (Citalopram, Escitalopram, Floxetine, Paroxetine and Sertraline) (13.51%), SNRI (Minalcipran & Venlafaxine) (8.98%), TCA (Amitriptyline, Clomipramine, Dosulepine, Imipramine) (3.16%)	**4 weeks after AD treatment** 4.8% experienced antidepressant-emergent manic switch. The risk of switching appeared to be significantly related to the cumulative lifetime number of mood episodes, in particular manic episodes.	Not applicable
Scott et al., 2017 [7]	A re-examination of antidepressant treatment-emergent mania in bipolar disorders: evidence of gender differences	Cross-sectional study	BD I or II patients (*n* = 210), ATEM + = 75); ATEM − = 135)	AD monotherapy (SSRIs = 46%; AD plus mood stabilizer	ATEM comprised 36% of the sample; 87% of ATEM occurred during ADM monotherapy (SSRIs = 46%, TCAs = 21%, SNRIs = 9%, other = 12%); ATEM differ significantly according to gender.	Not applicable
Amerio and Odone, 2018 [36]	Clinical management of comorbid bipolar disorder and obsessive-compulsive disorder: A case series	Longitudinal clinical observation	Three patients with obsessive-compulsive disorder	Sertraline 250 mg/day Citalopram 60 mg/day Clomipramine 150 mg/day	**Treatment SSRI and TCA** ↑ manic episodes	Not applicable
Fornaro et al., 2018 [8]	Clinical and psychopathological features associated with treatment-emergent mania in bipolar-II depressed outpatients exposed to antidepressants	Cross-sectional study	BD-II (*n* = 91; TEM+ = 35, TEM − = 56)	Antidepressants (including monotherapy, as well as infra- and inter-class combination treatment with multiple antidepressants)	TEM occurred in 38.46% of the assessed cases.	Not applicable
Berkol et al., 2019 [21]	Clinical characteristics of antidepressant use and related manic switch in bipolar disorder	Cross-sectional study	Euthymic BD patients (*n* = 238)	AD monotherapy and AD combination	47.15% of 123 patients who received ADs at least once had experienced a manic switch under AD treatment. AD use longer than 12 months was negatively associated with the occurrence of manic switch.	Not applicable
Koc et al., 2022 [55]	Association between thyroid autoimmunity and antidepressant treatment-emergent mania in pediatric mood disorders.	A cross-sectional study	29 antidepressant treatment-emergent mania patients (ATEM+); 31 psychiatric controls (ATEM-)	sertraline, fluoxetine, escitalopram, venlafaxine	ATEM+ group was mostly treated with a combination of antidepressants and antipsychotics	Seroprevalence and titer of TPO-abs in patients with pediatric ATEM + and ATEM- controls (51,7% vs. 22,6%). Our results demonstrate that seroprevalence and titer of TPO-abs are significantly higher in ATEM+ than in ATEM-; TPO-abs positivity was significantly associated with ATEM+
Gardea-Resendez et al., 2023 [54]	Antidepressants that increase mitochondrial energetics may elevate risk of treatment-emergent mania.	Cross-sectional study	BD patients phenotyped as TEM+ (*n* = 176) or TEM- (*n* = 516)	bupropion, nortriptyline, paroxetine, and venlafaxine	AD Mito+ ↑ TEM compared to antidepressants that decreased this activity (Mito−): Mito + 24.7% vs. Mito − 13.5%; OR = 2.21; *p* = 0.000009	Not applicable
Jefsen et al., 2023 [60]	Revisiting the association between treatment with antidepressants and mania: A nationwide within-individual study of 3554 patients with bipolar disorder	Within-individual observational study	BD patients (*n* = 3554)	Antidepressant (ATC code: NA06)	The number of manic episodes peaked approximately 3 months prior to initiation of antidepressant treatment, and the number of depressive episodes peaked around the initiation of antidepressant prescription.	Not applicable
Joas et al., 2023 [39]	Effect of CYP2C19 polymorphisms on antidepressant prescription patterns and treatment emergent mania in bipolar disorder.	Swedish bipolar cohort collection (SWEBIC); genetic study of bipolar disorder	BD patients (*n* = 5019) 62% were women, and 44% had BD type I	20 mg citalopram, 10 mg escitalopram, 50 mg sertraline, 100 mg clomipramin, and 75 mg mitriptyline	Slower metabolism had higher risk of mania during the first 3 months of sertraline treatment (HR 1.3, 95% CI 1.04–1.62, *p* = 0.020) and amitriptyline or clomipramine treatment (HR 1.46, 95% CI: 1.05–2.02, *p* = 0.024)	Association between CYP2C19 metabolic phenotypes and a manic episode within 3 months of starting treatment with either sertraline or the tricyclic antidepressants amitriptyline and clomipramine
Nuñez et al., 2024 [28]	Pharmacogenomic overlap between antidepressant treatment response in major depression & antidepressant associated treatment emergent mania in bipolar disorder	Genome-wide association study	Adult BD patients (*n* = 861)	SSRI and SNRI	Patients with TEM were more likely to be female, have BD-I, and, have a higher rate of history of serious suicide attempts.	No difference in genome-wide significant variants. A higher polygenic score for antidepressant response in MDD was associated with higher odds of treatment emergent mania in BD-I.
Liu, Ting-Hui et al., 2025 [58]	Risk of manic switch and suicidal outcomes in bipolar depression treated with esketamine: A one-year retrospective cohort study of 2126 patients	Retrospective cohort study	Adult BD patients with bipolar depression (*n* = 2126)	Esketamine plus a mood stabilizer or an antipsychotic agent	Lower risk of manic switch associated with esketamine treatment at longer follow-up intervals. At longer follow-up intervals, female patients showed a significantly reduced risk of manic switch.	Not applicable
Liu, Shuang et al., 2025 [25]	Factors associated with the frequency of manic and depressive episodes in bipolar disorder: a multicenter study in China	Multicenter, longitudinal cohort study	Adult BD-I or BD-II subjects (*n* = 520)	AD exposure	The use of AD (OR = 1.52, *p* = 0.01) (and other factors) was associated with the number of manic episodes.	Not applicable
Chang et al., 2025 [57]	Antidepressant initiation and the risk of treatment-emergent mania in children and adolescents with depression: A real-world cohort study	Retrospective cohort study	MDD patients, aged 6–17 years (*n* = 105,728)	AD exposure	Non-robust association between antidepressant initiation and short-term risk of treatment-emergent mania.	Not applicable
Baldessarini et al., 2025 [56]	Responses to clinical treatment of bipolar versus unipolar depressive episodes in women versus men	Cross-sectional study	BD patients (*n* = 253) MDD patients (*n* = 2990)	AD monotherapy or AD combined with mood stabilizers, antipsychotics, and/or psychotherapy	2.0% of patients (92.3% BD subjects) switched from depression into (hypo)mania.	Not applicable
Dai et al., 2025 [49]	Clinical efficacy and safety of vortioxetine as an adjuvant drug for patients with bipolar depression	Prospective, two-center, randomized, 12-week pilot study	BD-II adult subjects in depressive episode (*n* = 100)	Lurasidone plus Vortioxetine or lurasidone plus valproate	Three subjects (10.3%, 3/29) treated with the combination of lurasidone and vortioxetine switched to mania.	Not applicable
Vöhringer et al., 2025 [59]	Antidepressant Remission and Manic Switch in Bipolar Depression: A Propensity Score Analysis	Follow-up cohort (2 years)	BD subjects in depressive episode (*n* = 2166, 1475 AD-exposed and 691 AD-unexposed)	AD exposure (concomitant treatments not examined)	461 (21.3%, 289 [19.6%] AD exposed and 172 [24.9%] AD unexposed) of patients switched for a manic (*n* = 106; 4.9%), hypomanic (*n* = 185; 8.5%), or mixed (*n* = 170; 7.8%) episode. No association between AD exposure and increased risk of manic switch No increase in the risk of manic switch over shorter follow-up periods of 90 days or 180 days in PS-adjusted models. ↑ risk of manic switch in AD-exposed subjects with past suicide attempts.	Not applicable
Hidalgo-Mazzei et al., 2026 [24]	Antidepressant-Associated Risk of Manic and Mixed Episode Hospitalization in Bipolar I Disorder: A 10-Year Population-Based Cohort Study	Retrospective cohort study	BD-I patients (*n* = 7946; 3973 AD users and 3973 AD non-users)	AD monotherapy (41.4%) AD plus antipsychotic (25.1%) AD plus mood stabilizer (12.9%) AD plus antipsychotic and mood stabilizer (20.6%)	6.3% of subjects experienced hospitalization for manic or mixed episodes. **AD use** ↑ risk of a manic/mixed episode hospitalization, with the highest risk for AD monotherapy. ↑ risk of mood switching during AD monotherapy for younger patients All treatment strategies were associated with increased risk of mixed episodes.	Not applicable
Huang et al., 2026 [29]	Genetic and clinical characteristics associated with antidepressant-induced mania in depression patients	Genome-wide association study	Depression patients (*n* = 772)	AD exposure	145 (19.65%) subjects presented antidepressant-induced mania within the 7- to 28-day treatment period. Female gender, postpartum depression, obsessive-compulsive disorder, severe depression, all substance use disorders, and other non-organic psychoses were associated with increased likelihood of antidepressant-induced mania.	8 SNPs (rs76303898, rs62290372, rs200932609, rs13382259, rs28712187, rs117072317, rs10744764, and rs544429574) were identified as potential genetic markers associated with antidepressant-induced mania.

*AD* antidepressants, *ATC* Anatomical Therapeutic Chemical, *ATEM* + Antidepressant treatment-emergent mania, *BD* bipolar disorder, *HR* hazard ratio, *MDD* major depressive disorder, *PS* propensity score, *SGA* second-generation antipsychotic, *SNPs* single nucleotide polymorphisms, *SNRI* serotonin and norepinephrine reuptake inhibitors, *SSRI* selective serotonin reuptake inhibitors, *TCA* tricyclic antidepressants, *TEM* treatment-emergent mania. ↑ Increase in the parameter; ↓ Decrease in the parameter

## Methods

The present study is a narrative review of scientific articles published in peer-reviewed journals between 2014 and 2026. Literature searches were conducted in PubMed using the following terms: (“mania“[MeSH]) AND (“antidepressive agents“[MeSH]); (manic switch[Title/Abstract]); (“antidepressive agents“[MeSH]); (“treatment-emergent mania/hypomania“[MeSH]). Only articles published in English were included. After the removal of duplicates and the exclusion of narrative and/or systematic reviews, as well as studies deemed irrelevant to the scope of this review, a total of 25 studies were selected for inclusion (a summary of the included studies is presented in Table 1 and 2). Additionally, other relevant articles were consulted to support the background and discussion of the findings.

## The Clinical Guidelines

The clinical management of BD remains a substantial challenge, requiring multimodal interventions and reflecting high rates of therapeutic non-adherence [14]. First-line long-term treatment primarily involves mood stabilizers such as lithium and anticonvulsants (e.g., valproate and lamotrigine), as well as atypical antipsychotics (e.g., quetiapine, aripiprazole, and cariprazine), which are recommended for both acute and maintenance therapy [15, 16]. Depressive symptoms — which are more prevalent than manic, hypomanic, or mixed episodes — often include persistent sadness, anhedonia, changes in sleep and appetite, psychomotor disturbances, and suicidal ideation [14, 17, 18]. Although these episodes share clinical features with major depressive disorder (MDD), their treatment differs significantly [19].

Clinical guidelines recommend lithium and lamotrigine as first-line treatments for acute bipolar depression, with quetiapine, lamotrigine, and lurasidone preferred as monotherapies [20]. Antidepressants may be used in combination with mood stabilizers or antipsychotics, particularly for BD type II; however, they are not recommended as monotherapy for long-term treatment [14, 21]. In addition to their limited efficacy, antidepressants have been associated with an increased risk of mood destabilization, affective switching, number of manic episodes, hospitalizations, and accelerated mood cycling [14, 21–25]. These risks are particularly elevated in individuals with BD type I, females, those with a history of severe suicide attempts, concomitant mental illness, and individuals with frequent prior manic episodes [23, 24, 26–29]. The neurobiological mechanisms underlying antidepressant-induced manic switching are not yet fully understood. Therefore, a clear understanding of how each class of antidepressants influences the risk of manic switching is clinically useful.

## Tricyclic Antidepressants and TEM

The association between tricyclic antidepressants (TCAs) and manic switching in BD is well-established [13, 27, 30], although some controversy remains [31]. A preclinical study demonstrated that imipramine could induce mania-like behavior in rats pretreated with ouabain (OUA) [32]. In this model, the OUA administration led to a mania-like state by the 7th day, followed by a depressive-like state by the 14th day. Notably, imipramine administration triggered mania-like behavior on the 14th-day post-OUA injection, mimicking a switch from depression to mania. At the molecular level, imipramine exacerbated OUA-induced oxidative damage in the rat brain, suggesting a potential synergistic interaction between imipramine and OUA [32].

**Fig. 1:**
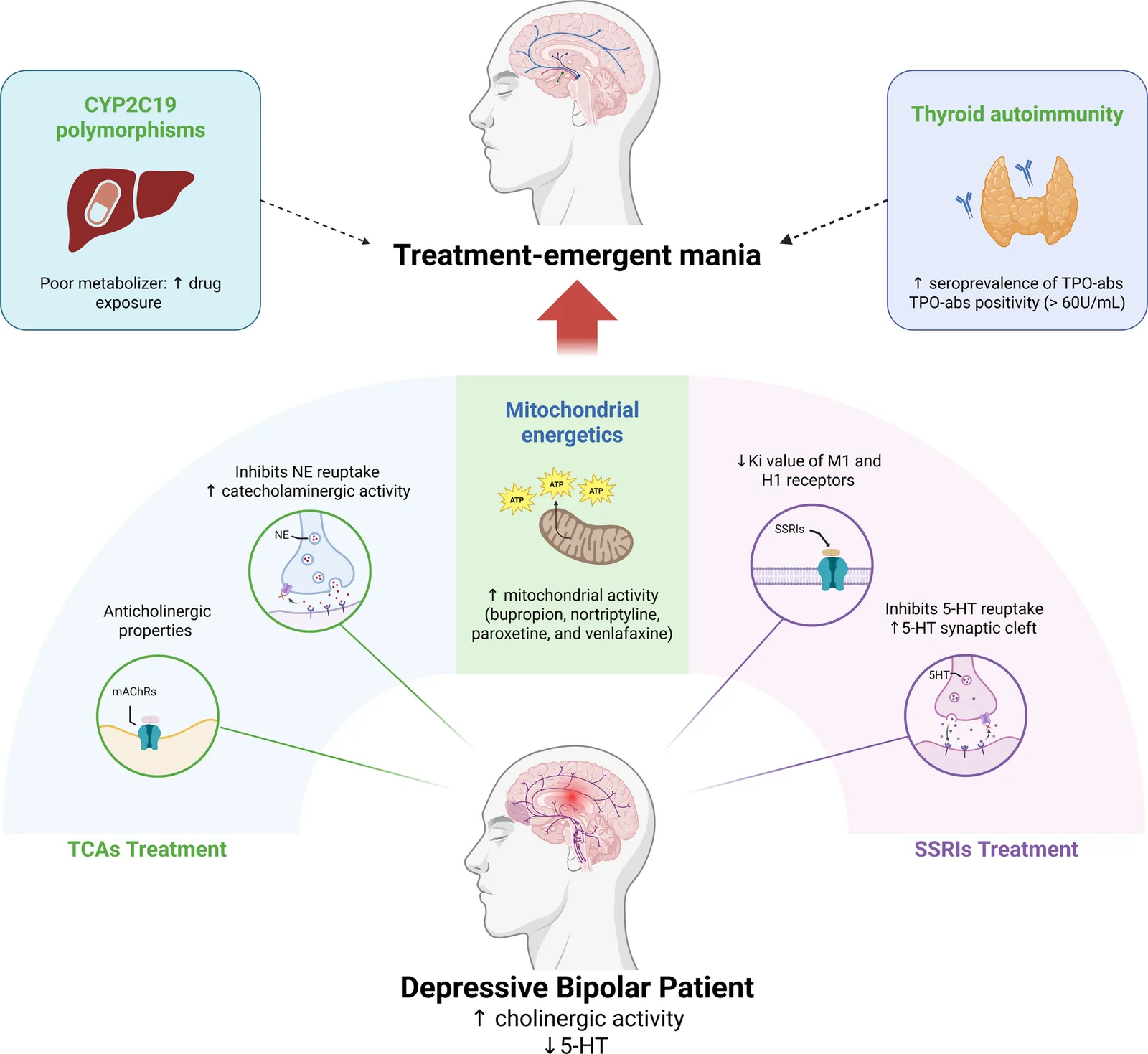
Mechanisms by which antidepressants may induce TEM. Tricyclic antidepressants are commonly associated with this phenomenon due to their anticholinergic properties, which involve the blockade of muscarinic receptors in limbic circuits. Additionally, they increase catecholaminergic neurotransmission by inhibiting the reuptake of serotonin and norepinephrine. Selective serotonin reuptake inhibitors may also trigger a manic switch by increasing the availability of serotonin in the synaptic cleft. Their interaction with muscarinic and histaminic receptors, as indicated by their low Ki values, may contribute to this effect. The risk of TEM can also be driven by pharmacogenetic, mitochondrial, and immunological factors. Ki = inhibitory constant; mAChRs = Muscarinic acetylcholine receptors; NE = Norepinephrine; SSRIs = Selective serotonin reuptake inhibitors; TCAs = Tricyclic antidepressants; TPO-abs = anti-thyroid peroxidase antibodies; 5-HT = Serotonin;. Created in BioRender. Valvassori, SS. (2026) https://BioRender.com/qkguq5u

In clinical settings, a cohort study of patients with bipolar depression (*n* = 1,242) found that 4.8% experienced a TEM within four weeks of treatment, including those receiving TCAs such as imipramine [27]. Compared to non-tricyclic antidepressants, TCAs — such as imipramine, amitriptyline, clomipramine, and dosulepine — were associated with more than double the risk of induced TEM in patients with bipolar depression [32–35], as well as individuals with obsessive-compulsive disorder [36]. The neurobiological mechanisms underlying this phenomenon are thought to involve disruptions in neurotransmitter balance (Fig. 1). Evidence for neuroimaging, neuropharmacological intervention, and genetic association studies suggests that increased cholinergic activity contributes to depressive symptoms, while elevated catecholaminergic activity (dopamine and norepinephrine) is associated with mania [37, 38]. The heightened risk associated with TCAs has been hypothesized to be caused by their anticholinergic properties (via muscarinic receptor blockade), which may disrupt the cholinergic-adrenergic balance in limbic pathways [35, 38, 39]. Additionally, TCAs strongly inhibit norepinephrine reuptake via the norepinephrine transporter (NET), thereby increasing synaptic norepinephrine levels [37]. This excess noradrenergic activity may destabilize monoaminergic balance and contribute to the risk of a manic switch. Moreover, dopaminergic effects of elevated norepinephrine (via crosstalk) may further exacerbate this risk [38].

Recent evidence suggests that hepatic metabolism may also modulate the risk of TEM. Joas and colleagues (2023) investigated the relationship between CYP2C19-predicted metabolism and antidepressant use, including TCAs, in BD. The authors evaluated two single-nucleotide polymorphisms (SNPs), rs4244285 and rs12248560, of the CYP2C19 enzyme. Regarding TCAs (such as amitriptyline 75 mg and clomipramine 100 mg), poor metabolizer phenotypes were associated with a higher risk of TEM (hazard ratio [HR] = 1.46). Furthermore, patients with an ultrarapid metabolizer phenotype had a higher risk of early treatment non-persistence (HR = 2.54). CYP2C19 metabolizes amitriptyline and clomipramine into their respective active metabolites, nortriptyline and desmethylclomipramine [39]. Slower metabolism of CYP2C19, with consequent higher drug exposure, may thus be a critical risk factor for TEM.

## Selective Serotonin Reuptake Inhibitor and TEM

Although less common than with TCAs, TEM has also been associated with selective serotonin reuptake inhibitor (SSRI) therapy [13, 30, 40]. A case series by Amerio et al. [36] reported that a 56-year-old woman with obsessive-compulsive disorder developed a manic episode after eight months of treatment with sertraline (200 mg/day). In another case, a 32-year-old man treated with citalopram (60 mg/day) developed a manic episode after three months. A multicenter study of bipolar patients (*n* = 1,720) found that a history of SSRI-induced switching, particularly fluoxetine, sertraline, and paroxetine, was associated with a higher risk of TEM [11]. Çiray et al. [40] reported that fluvoxamine treatment carried the highest risk of TEM (reporting odds ratio (OR): 13.957). Furthermore, a retrospective cohort study of 21,012 adults with depression (2006–2013) found that SSRI therapy was associated with an increased incidence of mania or BD, at a rate of 13.2 cases per 1,000 person-years [30].

In general, all SSRIs primarily inhibit serotonin reuptake in presynaptic neurons, thereby increasing the availability of serotonin (5-HT) to bind to postsynaptic receptors. Evidence suggests that 5-HT neurotransmission is involved in the pathophysiology of both depressive and manic episodes (typically decreased during depression and increased during mania) as well as in the mechanism of action of mood stabilizers [41, 42]. Some studies have also indicated that hypomanic and manic episodes are influenced not only by 5-HT levels but also by the modulation of specific 5-HT receptors, such as 5-HT1A, 5-HT1D, and 5-HT2 [43, 44]. However, the concomitant dysfunction of multiple monoaminergic systems is likely to further increase the risk of manic switch [38, 45].

A pharmacodynamic study suggested that one mechanism by which SSRIs may trigger mania involves their interaction with muscarinic (M1) and histamine (H1) receptors [40] (Fig. 1). Çiray et al. [40] reported that the inhibitory constant values for M1 and H1 receptors were inversely correlated with the risk of SSRI-induced TEM, accounting for 98% of the observed variance. Interestingly, serotonergic, noradrenergic, and dopaminergic receptors did not significantly influence this risk [40]. Nevertheless, the mechanisms through which the muscarinic and histaminergic systems modulate mood are complex and remain incompletely understood.

Regarding SSRIs such as sertraline, the previously cited study by Joas and colleagues identified an association between TEM and slow CYP2C19 metabolism (HR = 1.33, 95% CI 1.05–1.69) [39]. This result suggests a genetically mediated dose-response relationship, in which reduced CYP2C19 enzymatic activity leads to increased systemic drug exposure, which in turn raises the probability of TEM. These findings indicate that, in many cases, TEM is a pharmacokinetic phenomenon and differs significantly between sexes [7]. Since neurotransmitter regulation in BD is inherently unstable, the increase in monoamine availability within the synaptic cleft is only tolerated if maintained within physiological limits [39, 46]. From this perspective, it is hypothesized that in patients with CYP2C19 deficiency, the actual drug exposure in brain tissue is substantially higher than the prescribed dose [39]. This overexposure may overwhelm the central nervous system’s compensatory mechanisms, triggering TEM.

## Other Antidepressants and TEM

While the potential for TEM with monoamine oxidase inhibitors (MAOIs) and serotonin-norepinephrine reuptake inhibitors (SNRIs) is well-documented, our search found few or no studies specifically addressing these classes between 2014 and 2026. One study reported TEM associated with SNRI use, with 33.5% of participants having a history of manic switching [28]. In this genome-wide association study, Nuñez et al. found that polygenic scores — used to estimate how an individual’s genetic profile predicts disease risk — can also forecast antidepressant response in MDD. Notably, this genetically predicted response was associated with an increased risk of TEM in BD, particularly in BD type I [28]. These findings suggest that individuals with BD type I may have both a higher clinical and genomic susceptibility to TEM.

Regarding atypical antidepressants, a case report documented a TEM associated with agomelatine [47]. A 31-year-old female patient with unipolar depression developed manic symptoms after two months of treatment with agomelatine (25 mg/day). The clinical presentation included increased self-confidence, pressured speech, episodic irritability, irregular eating patterns, and a reduction in sleep duration to four hours per night. Additionally, the patient exhibited compulsive cleaning and excessive spending behaviors. The symptoms were resolved following discontinuation of agomelatine. Agomelatine has a unique pharmacological profile, acting as a selective melatonin (MT1/MT2) receptor agonist and a 5-HT2C receptor antagonist, with no significant affinity for monoamine transporters (SERT, NET, DAT) or GABAergic receptors [48]. This case highlights that even antidepressants with non-monoaminergic mechanisms may trigger manic switches in vulnerable individuals.

Multimodal antidepressants have also been investigated in bipolar depression. In one study, three of 29 participants receiving vortioxetine in combination with lurasidone developed a manic switch [49]. Vortioxetine is a multimodal serotonergic agent that acts as a 5-HT1A receptor agonist, a 5-HT1B partial agonist, and antagonist at 5-HT3, 5-HT7, and 5-HT1D receptors, in addition to inhibiting SERT [50]. Beyond its primary serotonergic effects, evidence suggests downstream modulation of additional neurotransmitter systems, including norepinephrine, dopamine, acetylcholine, histamine, glutamate, and GABA [51–53]. Collectively, these findings highlight the need for further controlled studies to clarify the safety and risk of manic switching associated with vortioxetine as an adjunctive treatment for bipolar depression.

Different antidepressants may influence mitochondrial bioenergetics in distinct ways. According to Gardea-Resendez et al. [54], antidepressants associated with increased mitochondrial activity (Mito+), such as bupropion, nortriptyline, paroxetine, and venlafaxine, were associated with a higher frequency of TEM compared with antidepressants associated with reduced mitochondrial activity (Mito−), such as amitriptyline and escitalopram. The authors observed that exposure to Mito+ antidepressants was associated with an approximately twofold higher rate of TEM (24.7% vs. 13.5%; OR = 2.21). Although the exact mechanisms remain uncertain, the findings suggest that alterations in electron transport chain activity and oxidative phosphorylation may contribute to vulnerability to mania in patients with BD. When considered alongside the findings of Joas et al. [39] regarding enzymatic metabolism, these results reinforce the hypothesis that pharmacokinetic and bioenergetic factors may differentially influence antidepressant response in individuals with BD.

In addition to pharmacogenetic and bioenergetic factors, recent evidence suggests that immunological mechanisms may also contribute to vulnerability to TEM associated with antidepressants. According to Koc et al. [55], pediatric patients with BD who developed TEM exhibited higher seroprevalence and higher titers of anti-thyroid peroxidase antibodies (TPO-abs) compared to patients without a history of TEM. TPO-abs positivity was associated with a significantly increased risk of antidepressant-related TEM (OR = 3.67), independently of clinical and demographic factors. These findings reinforce the hypothesis that inflammatory and autoimmune processes may participate in the pathophysiology of antidepressant-induced TEM [8, 55]. The authors further suggest that markers of thyroid autoimmunity may help identify young BD patients at increased risk for ATEM.

## Additional Considerations

It is important to acknowledge that some studies have reported a weak or non-significant association between antidepressant use and TEM [56–59]. However, in at least one of these investigations, concomitant treatments, such as mood stabilizers, were not evaluated, limiting extrapolation of the findings to antidepressant monotherapy in BD [54]. Jefsen et al. [60] also suggest that TEM may be a natural course of the disorder. They identified that the peak number of manic episodes occurred approximately three months before the start of antidepressant treatment, and the number of depressive episodes peaked at the beginning of antidepressant prescription. The authors propose that the observed association between antidepressant use and TEM may reflect time-varying confounding by indication rather than a true causal relationship between antidepressant exposure and manic switching. In addition, esketamine administered in combination with a mood stabilizer has been suggested as a potential option for the treatment of bipolar depression, although the current evidence remains preliminary and requires further confirmation in larger, controlled studies [58]. Taken together, while emerging data suggest that the risk of TEM may not be uniformly elevated across all antidepressant strategies, the potential for mood destabilization should always be carefully considered when prescribing antidepressants for individuals with BD.

This concern is mirrored in the context of unipolar depression. Although the focus of this study was antidepressant-induced mania switching, antidepressant-associated hypomania represents a significant clinical challenge in this population, with incidence rates varying considerably, with reported rates ranging from 0.3% to 22.4%, depending on factors such as drug class and duration of clinical follow-up [61]. Antidepressant-associated hypomania manifests with greater prevalence in females, young patients, and those with a positive family history of bipolar disorder. Regarding pharmacotherapy, it is observed that broad-spectrum antidepressants—particularly TCAs and certain MAOIs—present a significantly higher risk of inducing hypomania when compared to more selective agents, such as SSRIs [61].

## Clinical Considerations for Antidepressant Use in BD

When to consider an antidepressant in BD: Antidepressants may be considered in selected patients with bipolar depression, particularly when depressive symptoms persist despite adequate mood stabilization. Current clinical guidelines support their cautious use as adjunctive therapy combined with mood stabilizers or atypical antipsychotics, especially in BD type II and in patients without a recent history of manic or mixed episodes [14, 20, 23]. Antidepressants with lower switch liability, such as SSRIs, are generally preferred over TCAs, which consistently show higher rates of TEM [13, 35]. Careful patient selection, gradual dose titration, and close monitoring during the first weeks of treatment are essential.

When not to consider an antidepressant in BD: Antidepressants should generally be avoided as monotherapy in BD, particularly in BD type I, due to the increased risk of mood destabilization and TEM [14, 21, 23, 24]. Their use may also be inappropriate in patients with a history of antidepressant-induced TEM, rapid cycling, mixed features, frequent manic episodes, severe suicidal behavior, or comorbid psychiatric conditions associated with increased switching vulnerability [8, 13, 24, 28]. TCAs and antidepressants with broader monoaminergic or activating profiles should be prescribed with particular caution because of their stronger association with manic switching [32–35]. Emerging evidence further suggests that pharmacogenetic susceptibility, mitochondrial bioenergetic alterations, and autoimmune markers may increase the likelihood of TEM in vulnerable individuals [39, 54, 55].

## Limitations

We acknowledge some limitations in this review. As it is a narrative review, rather than a systematic review or meta-analysis, which may increase the risk of selection bias. The included studies are heterogeneous in terms of design, population characteristics, diagnostic criteria, exposure to antidepressants, and definitions of TEM, which limits direct comparisons between studies. Furthermore, several conclusions derive from observational studies, case reports, or preclinical models, which restrict causal inference. Finally, some classes of antidepressants remain poorly explored in the recent literature, particularly MAOIs and SSRIs.

## Future Directions

The findings strengthen the argument that safe and effective treatment of bipolar depression requires a mechanistically informed, individualized approach that prioritizes mood stabilization and vigilant monitoring over antidepressant-centric strategies. Future research should therefore move beyond class-based risk generalizations and focus on: (i) identifying robust predictors of switching (genetic markers, clinical phenotypes, and biological signatures), (ii) clarifying how antidepressant-related perturbations propagate across cholinergic, adrenergic, serotonergic, and receptor-interaction networks to produce state transitions, and (iii) developing precision-treatment frameworks that can prospectively match patients to the lowest-risk interventions while preserving antidepressant efficacy where appropriate. Advancing this agenda is essential not only to reduce iatrogenic harm, but also to leverage antidepressant-induced switching as a window into the core neurobiology of mood instability—ultimately enabling more targeted, preventive, and personalized therapeutics for BD.

## Conclusion

Although antidepressants may contribute to mood destabilization in susceptible individuals, TEM is not a universal consequence of antidepressant exposure. The evidence reviewed here suggests that TEM is better conceptualized as a vulnerability-dependent phenomenon arising from interactions between antidepressant-related neurobiological effects and individual susceptibility factors, including BD subtype, prior illness course, pharmacogenetic variability, mitochondrial bioenergetics, and immunoinflammatory status. Tricyclic antidepressants remain the agents most consistently associated with switching risk, whereas SSRIs and atypical antidepressants may also precipitate TEM under specific neurobiological conditions. Collectively, these findings support a more nuanced and mechanistically informed understanding of antidepressant-associated TEM and reinforce the importance of individualized treatment strategies that prioritize mood stabilization, careful patient selection, and close clinical monitoring in the management of bipolar depression.

## Key References

- Hidalgo-Mazzei D, Anmella G, De Prisco M, Oliva V, Fico G, Valenzuela-Pascual C, et al. Antidepressant-Associated Risk of Manic and Mixed Episode Hospitalization in Bipolar I Disorder: A 10-Year Population-Based Cohort Study. Bipolar Disord. 2026;28:e70088. https://doi.org/10.1111/bdi.70088.
  - This reference provides evidence from a 10-year cohort study on how antidepressant use impacts the risk of hospitalization for manic or mixed episodes in BD type I.
- Çiray RO, Halaç E, Turan S, Tunçtürk M, Özbek M, Ermiş Ç. Selective serotonin reuptake inhibitors and manic switch: A pharmacovigilance and pharmacodynamical study. Asian J Psychiatr. 2021 Dec;66:102891. doi: 10.1016/j.ajp.2021.102891.
  - This study contributes to the understanding of manic switching because it proposes specific pharmacological mechanisms and receptors that may underlie this phenomenon in bipolar disorder. The authors report that the SSRI-induced manic switch is inversely correlated with the affinity of the drugs for muscarinic M1 and histamine H1 receptors.
- Joas E, Jonsson L, Viktorin A, Smedler E, Pålsson E, Goodwin GM, Landén M. Effect of CYP2C19 polymorphisms on antidepressant prescription patterns and treatment emergent mania in bipolar disorder. Pharmacogenomics J. 2023 Jan;23(1):28-35. https://doi: 10.1038/s41397-022-00294-4.
  - This study suggests that TEM is a biological event modulated by the individual's pharmacokinetics.
- Gardea-Resendez M, Coombes BJ, Veldic M, Tye SJ, Romo-Nava F, Ozerdem A, Prieto ML, Cuellar-Barboza A, Nunez NA, Singh B, Pendegraft RS, Miola A, McElroy SL, Biernacka JM, Morava E, Kozicz T, Frye MA. Antidepressants that increase mitochondrial energetics may elevate risk of treatment-emergent mania. Mol Psychiatry. 2023 Mar;28(3):1020-1026. https://doi:10.1038/s41380-022-01888-x.
  - This article focuses on the role of mitochondrial function in antidepressant-induced TEM. Antidepressants that increase mitochondrial energy production have been associated with a higher risk of inducing TEM in BD patients.
- Koc D, Ince E, San T, Akan P, Paketci A, Bober E, Tecirli ND, Inal N, Akay AP. Association between thyroid autoimmunity and antidepressant treatment-emergent mania in pediatric mood disorders. Psychiatry Res. 2022 Aug;314:114676. https://doi: 10.1016/j.psychres.2022.114676.
  - This study investigated the presence of antibodies (such as Anti-TPO), even when thyroid hormone levels (T3, T4, TSH) are within clinical normal limits. Patients with positive thyroid autoimmunity showed a significantly higher risk of developing antidepressant-associated TEM.
- Keramatian K, Chithra NK, Yatham LN. The CANMAT and ISBD Guidelines for the Treatment of Bipolar Disorder: Summary and a 2023 Update of Evidence. Focus (Am Psychiatr Publ). 2023;21:344–53. https://doi.org/10.1176/APPI.FOCUS.20230009.
  - This reference summarizes the latest evidence on bipolar disorder treatment, updating the CANMAT/ISBD guidelines, and is essential for psychiatric practice.

## Data Availability

No datasets were generated or analysed during the current study.
